# Effect of Preoperative Superselective Embolization in the Resection of Hypervascular Tumors of the Central Nervous System, Neck, and Nasal Cavity

**DOI:** 10.7759/cureus.102700

**Published:** 2026-01-31

**Authors:** Luis Abraham Castro Toscano, Diego Julian Alvis Peña, Jorge Antonio Morales Baez, Heidy Margarita Castro Lopez, Karen Eloisa Xochipa Ruiz, Fernando de Jesus Espinosa Lira, Gustavo Melo Guzmán

**Affiliations:** 1 Neurosurgery and Neurological Endovascular Therapy, Juárez Hospital of México, Mexico City, MEX

**Keywords:** esthesioneuroblastoma, hypervascular tumors of the central nervous system, juvenile nasopharyngeal angiofibromas, paragangliomas, preoperative embolization

## Abstract

Background

Hypervascular head and neck tumors, such as paragangliomas, esthesioneuroblastomas, and juvenile nasopharyngeal angiofibromas (JNA), are surgically challenging due to their rich vascular supply and proximity to critical structures. Preoperative superselective embolization may optimize surgical outcomes by reducing intraoperative bleeding and facilitating safe resections.

Methods

We conducted a retrospective case-control study of patients with hypervascular tumors of the central nervous system, neck, and nasal cavity treated between 2019 and 2024 at a single tertiary neurosurgical center. Patients were divided into embolization and non-embolization groups. Outcomes included the extent of resection, intraoperative blood loss, transfusion requirements, operative time, hospital stay, and complications.

Results

Fifty patients were analyzed (mean age 42.9 years; 58% female). Half underwent preoperative embolization, more frequently in nasal tumors (64%). Diagnoses included paraganglioma (46%), JNA (28%), and esthesioneuroblastoma (26%). Ethylene-vinyl alcohol copolymer was used in 96% of embolizations. Embolized patients had higher complete resection rates (100% vs. 84.6%; p<0.001), less blood loss (263.7 vs. 978.0 mL; p<0.001), fewer transfusions (16% vs. 84%; OR: 27.56), and shorter hospital stays (1.84 vs. 6.00 days; p<0.001). Complications were uncommon (8%) and transient.

Conclusion

Preoperative superselective embolization, particularly with ethylene-vinyl alcohol copolymer, is a safe and effective adjunct that improves resection rates, reduces perioperative morbidity, and optimizes surgical outcomes in hypervascular head and neck tumors.

## Introduction

Rare hypervascular tumors of the head and neck, such as paragangliomas, esthesioneuroblastomas, and juvenile nasopharyngeal angiofibromas (JNA), pose a considerable surgical challenge due to their complex anatomy, rich vascular supply, and potential for local invasion or metastatic dissemination [1,2]. Although their overall incidence is low, these tumors demand a meticulous diagnostic and therapeutic strategy to achieve optimal oncologic control while minimizing perioperative morbidity [3].

Paragangliomas are benign, slow-growing neuroendocrine neoplasms arising from parasympathetic paraganglia. They predominantly affect middle-aged women and, in hereditary cases, are frequently associated with germline mutations in the rearranged during transfection (RET), Von Hippel-Lindau (VHL) tumor suppressor gene, succinate dehydrogenase complex iron-sulfur subunit B (SDHB), and succinate dehydrogenase complex subunit D (SDHD) [4]. Esthesioneuroblastoma, originating from the olfactory neuroepithelium, accounts for approximately 3% of malignant nasal tumors and is characterized by locally aggressive behavior, with a propensity for cervical, pulmonary, and osseous metastases [5,6]. JNA, almost exclusively diagnosed in adolescent males, is histologically benign but locally aggressive, with intracranial extension reported in up to 30% of cases [5].

Within this framework, preoperative endovascular embolization has become an integral component of multidisciplinary management involving neurosurgery and otorhinolaryngology [1,2,4]. Its primary goal is selective devascularization of the tumor using particulate, liquid, or mechanical embolic agents, thereby reducing intraoperative blood loss and facilitating safe and complete resection [2,4]. Although complications are relatively uncommon, they may include cranial nerve deficits, cerebral ischemia, or inadvertent embolization via extracranial-intracranial anastomoses, highlighting the importance of detailed angiographic planning [1,3,5].

## Materials and methods

Study design and population

An observational, analytical, and retrospective case-control study was conducted at a single tertiary care hospital specializing in neurosurgery and neurological endovascular therapy (Juárez Hospital of Mexico). The study was reviewed and approved by the Institutional Review Board (Medical Specialties Thesis Protocol Subcommittee) of the Research and Teaching Division at Juárez Hospital of Mexico, under registration number HJM 175/24-R.

The study population included all patients, regardless of age or sex, diagnosed with hypervascular tumors of the central nervous system, neck, or nasal cavity, who underwent surgical resection between January 1, 2019, and December 31, 2024. Patients were grouped according to whether or not they received preoperative superselective embolization before tumor resection.

Inclusion and exclusion criteria

Patients were eligible if they had a confirmed diagnosis of hypervascular tumors, including paragangliomas, juvenile nasopharyngeal angiofibromas, or esthesioneuroblastomas, and underwent surgical resection with or without prior superselective embolization. Only patients with complete clinical, surgical, and follow-up records were included. 

Patients with incomplete medical histories or those who did not undergo surgical treatment were excluded.

Outcomes and data collection

The primary outcome was the effect of preoperative superselective embolization on the extent of tumor resection. Secondary outcomes included intraoperative blood loss, need for packed red blood cell transfusion, percentage of tumor resection, surgical time (in minutes), duration of hospital stay (in days), and procedure-related complications.

Demographic and clinical data were obtained through retrospective chart review. Variables included age, sex, tumor location (nasal or cervical), histological subtype, preoperative embolization status, intraoperative findings, and postoperative outcomes. Tumor staging was assessed using the Kadish classification for esthesioneuroblastomas [7], the Andrews-Fisch classification for juvenile nasopharyngeal angiofibromas [8], and the Shamblin classification for paragangliomas [9].

Preoperative embolization procedure

Preoperative embolization was performed 24 to 72 hours prior to surgery via superselective transarterial catheterization under fluoroscopic guidance. All procedures were carried out by neurosurgeons with subspecialty training in neurological endovascular therapy. The primary embolic agent used was ethylene-vinyl alcohol copolymer (EVOH), delivered through microcatheters targeting the dominant feeding arteries. The goal of embolization was to achieve maximal devascularization of the lesion while preserving the adjacent functional vasculature.

Statistical analysis

Descriptive and inferential statistical analyses were performed. Continuous variables were expressed as means, medians, and standard deviations. Frequency distributions were calculated for categorical variables. Measures of association, including odds ratios with 95% confidence intervals, were used to compare embolized and non-embolized groups. The Mann-Whitney U test was applied for nonparametric comparisons. A p-value ≤0.05 was considered statistically significant. Data analysis was performed using SPSS software, version 26.0 (IBM, Inc., Armonk, USA).

## Results

A total of 50 patients with hypervascular tumors of the central nervous system, neck, and nasal cavity were included in the study. Among them, 25 (50%) underwent presurgical superselective embolization. The mean age was 42.9±19.7 years (range: 6-82), with a predominance of female patients (58%, n=29). Embolization was more frequently performed in nasal tumors (64%, n=32), compared with cervical tumors (36%, n=18), reaching a significant difference (p=0.048).

The most frequent histological diagnoses were paraganglioma in 23 (46%) patients, juvenile nasopharyngeal angiofibroma in 14 (28%), and esthesioneuroblastoma in 13 (26%; see Tables 1 and 2). 

**Table 1:** Comparison of clinical and diagnostic characteristics by embolization in patients with head and neck tumors undergoing surgical resection Categorical variables were compared using the chi-square test. Age was compared using the Mann–Whitney U test. Confidence intervals (95% CI) are shown for each proportion or mean. A p-value ≤0.05 was considered statistically significant. EVOH -  ethylene-vinyl alcohol copolymer; n/a - not applicable

Variables, n (%), mean±SD (min, max)	Total	Embolization		p-value	OR (IC 95%)
	n=50	Yes, n=25	No, n=25		
Sex and age					
Female	29 (58.0%)	11 (44.0%)	18 (72.0%)	0.045	0.306 (0.094-0.992)
Male	21 (42.0%)	14 (56.0%)	7 (28.0%)		
Age	42.90±19.75 (6, 82)	40.20±20.81 (6, 81)	45.60±18.67 (11, 82)	0.339	n/a
Tumor location					
Nasal	25 (50.0%)	16 (64.0%)	9 (36.0%)	0.048	0.316 (1.00-1.004)
Neck	25 (50.0%)	9 (36.0%)	16 (64.0%)		
Diagnosis					
Paraganglioma	23 (46.0%)	9 (36.0%)	14 (56.0%)	0.154	0.44 (0.14-1.38)
Nasoangiofibroma	14 (28.0%)	10 (40.0%)	4 (16.0%)	0.059	3.5 (0.92-13.31)
Esthesioneuroblastoma	13 (26.0%)	6 (24.0%)	7 (28.0%)	0.718	0.79 (0.22-2.77)
Estesioneuroblastoma					
Kadish B	7 (53.8%)	3 (50.0%)	4 (57.1%)	0.68	0.75 (0.11-5.02)
Kadish C	4 (30.8%)	3 (50.0%)	1 (14.3%)	0.147	6 (0.64-56.15)
Kadish D	2 (15.4%)	0 (0.0%)	2 (28.6%)	0.353	0.27 (0.01-6.24)
Nasoangiofibroma					
Andrews Fisch I	2 (14.3%)	1 (10.0%)	1 (25.0%)	0.448	0.33 (0.02-6.62)
Andrews Fisch II	6 (42.9%)	5 (50.0%)	1 (25.0%)	0.333	3 (0.32-28.33)
Andrews Fisch IIIA	2 (14.3%)	1 (10.0%)	1 (25.0%)	0.448	0.33 (0.02-6.62)
Andrews Fisch IIIB	2 (14.3%)	2 (20.0%)	0 (0.0%)	0.441	2.69 (0.19-37.60)
Andrews Fisch IVA	2 (14.3%)	1 (10.0%)	1 (25.0%)	0.448	0.33 (0.02-6.62)
Paraganglioma					
Shamblin 1	2 (8.7%)	0 (0.0%)	2 (14.3%)	0.199	0.38 (0.02-9.36)
Shamblin 2	13 (56.5%)	6 (66.7%)	7 (50.0%)	0.437	2 (0.43-9.28)
Shamblin 3	8 (34.8%)	3 (33.3%)	5 (35.7%)	0.906	1.08 (0.24-4.96)
Embolization material					
EVOH	24 (96.0%)	24 (96.0%)	1 (4.0%)	<0.001	5.76 (3.15-10.53)
Microspheres	1 (4.0%)	1 (4.0%)	24 (96%)		

**Figure 1:**
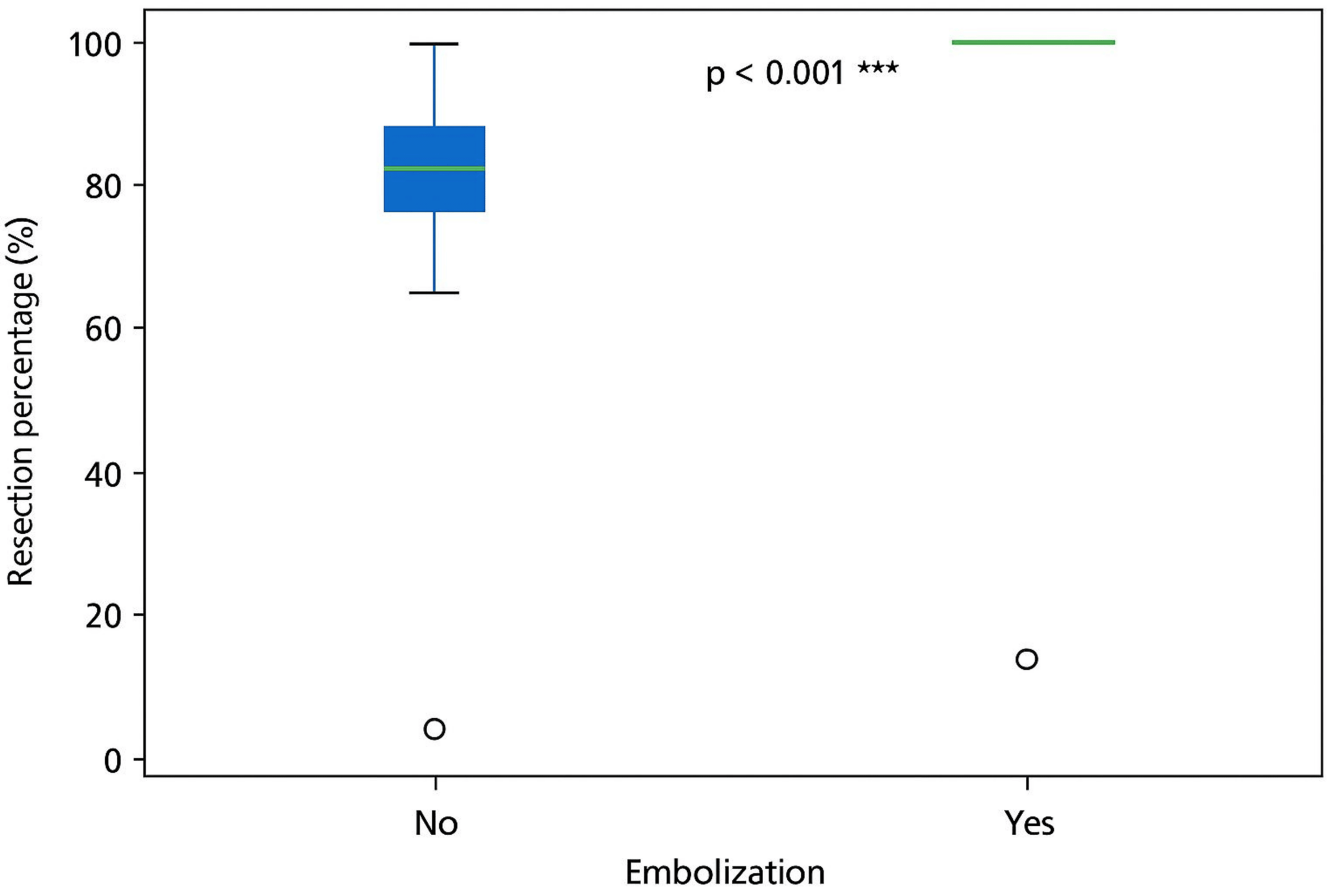
Extent of tumor resection (%) in embolized versus non-embolized patients with hypervascular head and neck tumors

**Figure 2:**
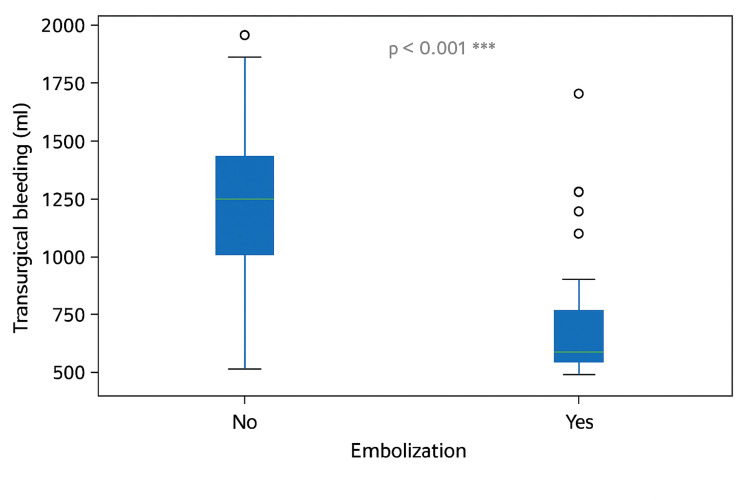
Intraoperative blood loss (mL) in embolized versus non-embolized patients with hypervascular head and neck tumors

Ethylene-vinyl alcohol copolymer (EVOH) was the most commonly used embolic agent, administered in 48 (96%) cases. Presurgical embolization was significantly associated with higher rates of complete tumor resection, 25, 100% vs. 1, 4.3% (p<0.001; see Figure 1); lower intraoperative blood loss, 263.7±389.8 mL vs. 978.0±494.4 mL (p<0.001; see Figure 2); reduced need for blood transfusion, 4, 16% vs. 21, 84% (p<0.001; OR: 27.56); and shorter postoperative hospital stays, 1.84±1.03 days vs. 6.00±3.20 days (p<0.001).

No statistically significant differences were observed between groups regarding surgical duration, tumor histological subtype distribution, or stage (see Tables 1-2). Postoperative complications were rare, occurring in 4 (8%) patients, including one case of cerebral embolism and one puncture-site hematoma (see Table 2).

**Table 2:** Surgical and postoperative outcomes according to embolization in patients with head and neck tumors undergoing surgical resection * Comparisons of continuous variables were performed using the Mann–Whitney U test Categorical variables were analyzed using the chi-square test. Confidence intervals (95% CI) and odds ratios (OR) are reported where applicable. A p-value ≤0.05 was considered statistically significant. n/a - not applicable

Variables, n (%), mean±SD (min, max)	Total	Embolization		p-value	OR (IC 95%)
	n=50	Yes, n=25	No, n=25		
Resection rate (%)	92.45±9.49 (70, 100)	100.00±0.00 (100, 100)	84.57±7.82 (70, 100)	<0.001*	n/a
Operating time (min)	152.82±75.49 (33, 350)	147.16±73.61 (33, 324)	158.48±78.42 (50, 350)	0.6011*	n/a
Intraoperative blood loss (ml)	620.84±569.45 (10, 2000)	263.68±389.75 (10, 1600)	978.00±494.36 (50, 2000)	<0.001*	n/a
Packed red cell transfusions (units)	0.80±1.01 (0.00, 4.00)	0.20±0.50 (0.00, 2.00)	1.40±1.04 (0.00, 4.00)	<0.001*	n/a
Postoperative hospital stay (days)	3.92±3.15 (1.00, 18.00)	1.84±1.03 (1.00, 4.00)	6.00±3.20 (2.00, 18.00)	<0.001*	n/a
Complications					
Absent	23 (92.0%)	23 (92.0%)	0	0.492	5.43 (0.25-11.73)
Cerebral embolism	1 (4.0%)	1 (4.0%)	0	1	n/a
Puncture site hematoma	1 (4.0%)	1 (4.0%)	0	1	n/a
Complete resection 100 %					
Yes	25 (53.2%)	24 (100.0%)	1 (4.3%)	<0.001	0.040 (0.006-0.273)
No	22 (46.8%)	0 (0.0%)	22 (95.7%)		
Need for transfusion					
Yes	25 (50.0%)	4 (16.0%)	21 (84.0%)	<0.001	27.563 (6.076-125.036)
No	25 (50.0%)	21 (84.0%)	4 (16.0%)		
Packed red cell transfusions (units)					
0	25 (50.0%)	21 (84.0%)	4 (16.0%)	<0.001	0.057 (0.014-0.233)
1	14 (28.0%)	3 (12.0%)	11 (44.0%)		
2	9 (18.0%)	1 (4.0%)	8 (32.0%)		
4	2 (4.0%)	0 (0.0%)	2 (8.0%)		

## Discussion

Preoperative superselective embolization of hypervascular head and neck tumors, including paragangliomas, juvenile nasopharyngeal angiofibromas, and esthesioneuroblastomas, constitutes an essential adjunctive strategy that facilitates safer and more complete resections while minimizing the risks associated with their abundant vascularization and proximity to critical neurovascular structures [10,11].

In this cohort of 50 patients treated at the Juárez Hospital of Mexico, presurgical embolization was significantly associated with lower intraoperative blood loss, reduced transfusion requirements, higher rates of total tumor resection, and shorter postoperative hospital stays. Notably, 100% of embolized patients achieved complete resection compared to 84.6±7.8% in the non-embolized group (p<0.001), findings that may translate into lower recurrence rates, decreased need for adjuvant therapies, and improved long-term outcomes.

These results are consistent with prior international studies demonstrating that preoperative devascularization enhances surgical field visualization, limits manipulation of adjacent critical structures, and decreases intra- and postoperative morbidity [12]. Reports by Iampreechakul et al., Artega et al., and Rosenbaum-Halevi et al. have similarly emphasized that embolization substantially improves resection control and visualization in tumors with extension into the skull base or orbit [13-15]. 

Histopathological analysis in this study revealed a predominance of paragangliomas among non-embolized patients, whereas juvenile nasopharyngeal angiofibromas were more frequent in the embolized group. Although these differences did not reach statistical significance, embolization was also employed in advanced tumor stages, such as Andrews-Fisch IIIB and IVA nasopharyngeal angiofibromas [16] and Kadish B-C esthesioneuroblastomas [16,17], allowing safer and more effective resections even in complex anatomical regions [17].

Ethylene-vinyl alcohol copolymer (EVOH; Onyx**™**) was used as the primary embolic agent in 96% of cases, reflecting its favorable handling characteristics, including non-adhesive behavior, controlled and gradual injection, deep tumor penetration, and the capacity for intermittent delivery that reduces the risk of non-target embolization [18]. The overall complication rate remained low, limited to two transient events without permanent sequelae. As emphasized in the literature, achieving optimal results depends on meticulous angiographic planning, precise microcatheterization, and appropriate embolic agent selection to mitigate risks of inadvertent embolization, tumor edema, or cranial nerve injury [19].

Collectively, these findings support the role of preoperative superselective embolization, particularly with EVOH, as a safe, effective, and technically advantageous intervention that enhances surgical outcomes in hypervascular head and neck tumors. By reducing intraoperative bleeding, transfusion requirements, and hospital stay while increasing complete resection rates, this approach contributes to improved perioperative safety and long-term tumor control [20,21].

The main limitations of this study include its retrospective design and single-center setting, which may introduce selection bias and restrict the external generalizability of the findings. The relatively small cohort limits statistical power for detecting subtle associations and performing subgroup analyses. Group allocation was non-randomized and likely influenced by clinical judgment, resulting in baseline imbalances in sex and tumor location that could act as confounders. Additionally, the near-exclusive use of EVOH precludes comparison with alternative embolic materials, preventing definitive conclusions about their relative efficacy.

## Conclusions

This study supports the role of preoperative superselective embolization as a valuable adjunct in the surgical treatment of hypervascular tumors of the head, neck, and central nervous system. By improving the intraoperative surgical field and reducing technical complexity, embolization contributes to safer and more effective resections. The results suggest that patients who undergo embolization are more likely to achieve complete tumor removal, require fewer transfusions, and experience shorter postoperative recovery times, with minimal complication rates. These findings highlight the clinical utility of embolization in enhancing surgical outcomes, particularly in anatomically challenging or richly vascularized tumors. Although the study's retrospective design and limited sample size prevent definitive conclusions, the consistency of the observed benefits reinforces the potential of embolization as a standard preoperative strategy in selected cases. Future prospective and multicenter research will be essential to validate these outcomes and further clarify patient selection criteria and long-term prognostic impact.
